# Dendritic cell tumor in a salivary gland lymph node: a rare differential diagnosis of salivary gland neoplasms

**DOI:** 10.1186/1746-1596-6-94

**Published:** 2011-09-30

**Authors:** Sebastian P Schraven, Stefan K Plontke, Roland Syha, Falko Fend, Hartwig Wolburg, Patrick Adam

**Affiliations:** 1 Department of Otolaryngology, Head and Neck Surgery, University of Tübingen, Elfriede-Aulhorn-Straße 5, D-72076 Tübingen, Germany; 2 Department of Otolaryngology, Head and Neck Surgery, University of Halle-Wittenberg, Magdeburger Straße 12, D-06112 Halle (Saale), Germany; 3 Department of Diagnostic and Interventional Radiology, University of Tübingen, Hoppe-Seyler-Straße 3, D-72076 Tübingen, Germany; 4 Institute of Pathology and Comprehensive Cancer Center (CCC), University of Tübingen, Liebermeisterstraße 8, D-72076 Tübingen, Germany; 5 University of Würzburg, Department of Otolaryngology, Head and Neck Surgery, Josef-Schneider-Strasse 11, 97080 Würzburg, Germany

**Keywords:** Dendritic cell tumor, salivary gland lymph node

## Abstract

Dendritic cell tumors are extremely rare neoplasms arising from antigen-presenting cells of the immune system. We report a case of a 69-year-old man with an unremarkable medical history who presented with a 2-months history of a gradually enlarging painless, firm, mobile, 2 × 2-cm swelling at the caudal pole of the left parotid gland without systemic symptoms. Histologically, the tumor consisted of a spindle cell proliferation in an intraparotideal lymph node. Based on the histopathologic, immunohistochemical and electron microscopic findings, a dendritic cell tumor, not otherwise specified (NOS) in an intraparotideal lymph node was diagnosed.

The patient underwent complete tumor resection, and is currently free of disease, 2 years after surgery. These extremely rare tumors must be distinguished from other more common tumors in the salivary glands. Awareness that dendritic cell tumors may occur in this localization, careful histologic evaluation and ancillary immunohistochemical and electron microscopical analyses should allow for recognition of this entity.

## Background

Dendritic cell sarcomas (DCS) are exceedingly rare entities, arising from antigen-presenting cells of the immune system. DCS are subclassified into the better characterized follicular (FDCS) [1] and interdigitating (IDCS) [2] dendritic cell sarcomas and other rare and less well classifiable dendritic cell tumors like fibroblastic reticular cell tumors, indeterminate dendritic cell tumors and dendritic cell tumors, not otherwise specified (DCT, NOS) [2]. DCS was first described in 1986 by Monda et al. [3]. Since then, nearly 300 cases, most of them FDCS, have been described in the literature. Although most DCS evolve in cervical, mediastinal, axillary and inguinal lymph nodes, also extranodal manifestations have been described [4]. The clinical behaviour of DCS is similar to that of low-grade soft tissue sarcoma, with an approximately 30% overall risk for developing local recurrences and metastases [5]. Because of the rareness of the disease a standardized treatment is lacking.

We herein report a case of a dendritic cell tumor, NOS of an intraparotideal lymph node, emphasizing the important role of ancillary immunohistochemical and molecular studies in establishing this extraordinarily rare diagnosis.

## Case report

A 69-year-old man presented with a 2-months history of a gradually enlarging painless, firm, mobile, 2 × 2 × 2 cm swelling at the caudal pole of the left parotid gland without systemic symptoms. His medical history was unremarkable.

**Figure 1:**
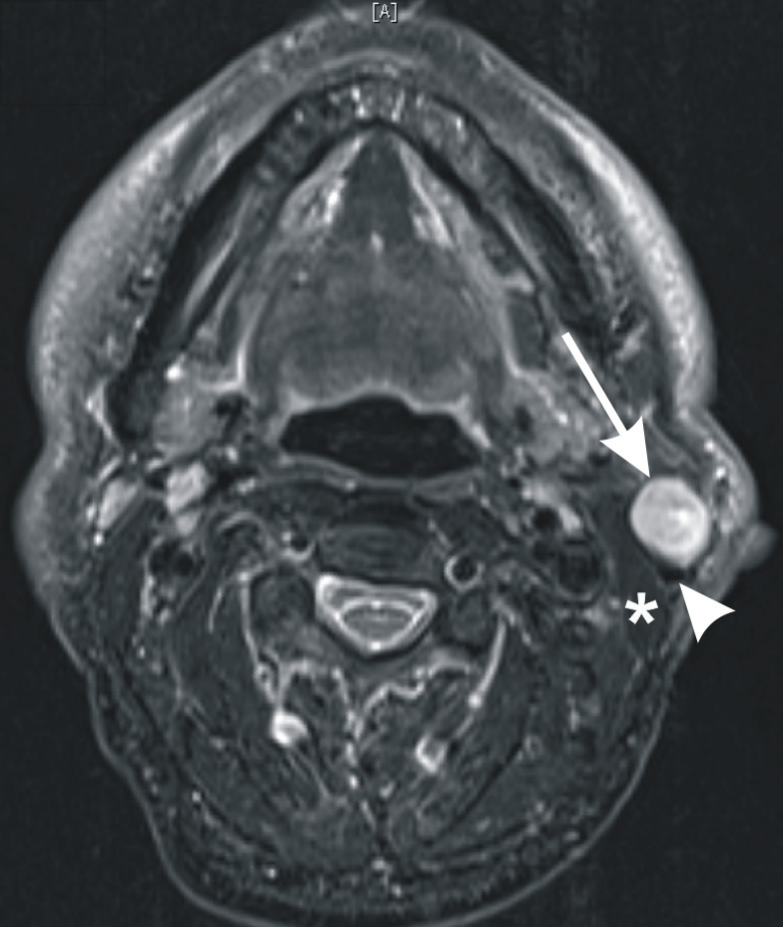
**Axial MR image**. (T2-weighted) of a 2 × 2 cm mass with hyperintense signal (arrow) directly bordering the lateral part of left sternocleidomastoid muscle (asterisk) and displacing the extern jugular vein (arrow head) dorsally. Cranially there was no clear demarcation to the left parotid gland.

Magnet resonance imaging (MRI) showed a 2 × 2 × 2 cm mass with hyperintense signal on T2-weighted images and hypointense signal on T1-weighted sequences and a contrast enhancement which bordered directly to the lateral part of left sternocleidomastoid muscle and displaced the external jugular vein dorsally. Cranially there was no clear demarcation to the left parotid gland (Figure 1). The patient underwent surgical excision of the swelling by a partial left parotidectomy with preservation of the facial nerve. Because of insecure R0-status a follow-up resection with extended partial parotidectomy and ipsilateral selective neck dissection (levels II and III) was conducted. A primary tumor of the upper aerodigestive tract was excluded by panendoscopy. Subsequent total body positron emission tomography with 18-F-fluorodesoxyglucose and computed tomography scan 6 months after surgery were unremarkable. The patient is currently disease free, 2 years after surgery.

## Material and methods

The specimen was fixed in 10% buffered formalin, paraffin-embedded, and histologic sections were obtained. Sections were routinely stained with hematoxylin and eosin. Immunohistochemical staining was performed on formalin-fixed, paraffin-embedded tissue sections on an automated immunostainer (Ventana Medical Systems^©^, Tucson AZ) following the manufacturer's protocols. The monoclonal antibodies used are listed in Table 1.

**Table 1:** List of antibodies and staining results.

Antibody	Company	Dilution	Tumor cells
Vimentin	DAKO	1:1000	**positive**

HLA-DR	DAKO	1:200	**part.positive**

Fascin	Abcam	1:100	**positive**

Langerin	eBioscience	1:50	negative

Lysozym	DAKO	1:400	negative

S100	DAKO	1:5000	negative

AE1/3	DAKO	1:100	negative

HMB45	DAKO	1:50	negative

MITF	Zytomed	1:100	negative

PMZ	Zytomed	1:50	negative

LCA	DAKO	1:500	negative

Calponin	DAKO	1:200	negative

Caldesmon	DAKO	1:50	negative

Clusterin	Menarini	1:30	negative

EMA	DAKO	1:100	negative

SMA	DAKO	1:500	negative

CK5/6	DAKO	1:100	negative

CK8/18	Zytomed	1:100	negative

OSCAR	Zytomed	1:200	negative

Synaptophysin	DCS	1:200	negative

Chromogranin	DAKO	1:500	negative

Factor XIIIa	DCS	1:100	negative

CD1a	DAKO	1:50	negative

CD3	Santa Cruz	1:200	negative

CD4	Zytomed	1:200	negative

CD20	DAKO	1:500	negative

CD21	Zytomed	1:50	negative

CD23	Novocastra	1:30	negative

CD30	DAKO	1:50	negative

CD31	DAKO	1:100	negative

CD34	DAKO	1:50	negative

CD35	Novocastra	1:50	negative

CD56	Cell Marque	1:100	negative

CD68 (PG-M1)	DAKO	1:200	negative

CD117	DAKO	1:200	negative

CD123	BD Pharmingen	1:50	negative

Mib1	DAKO	1:200	40-50%

For electron microscopical (EM) evaluation, small pieces of tissue were dissected out of the paraffin bloc, rehydrated stepwise and postfixed in 1% OsO_4 _in 0.1 M cacodylate buffer (pH 7.4) and then again dehydrated in an ethanol series (50, 70, 96, 100%). The 70% ethanol was saturated with uranyl acetate for contrast enhancement. Dehydration was completed in propylene oxide. The specimens were embedded in Araldite (Serva^©^, Heidelberg, Germany). Ultrathin sections were produced on a FCR Reichert Ultracut ultramicrotome (Leica^©^, Bensheim, Germany), mounted on pioloform-coated copper grids, contrasted with lead citrate and analyzed and documented with an EM10A electron microscope (Carl Zeiss^©^, Oberkochen, Germany). The pictures were digitized and processed with Adobe Photoshop.

## Results

### Gross and histological findings

**Figure 2:**
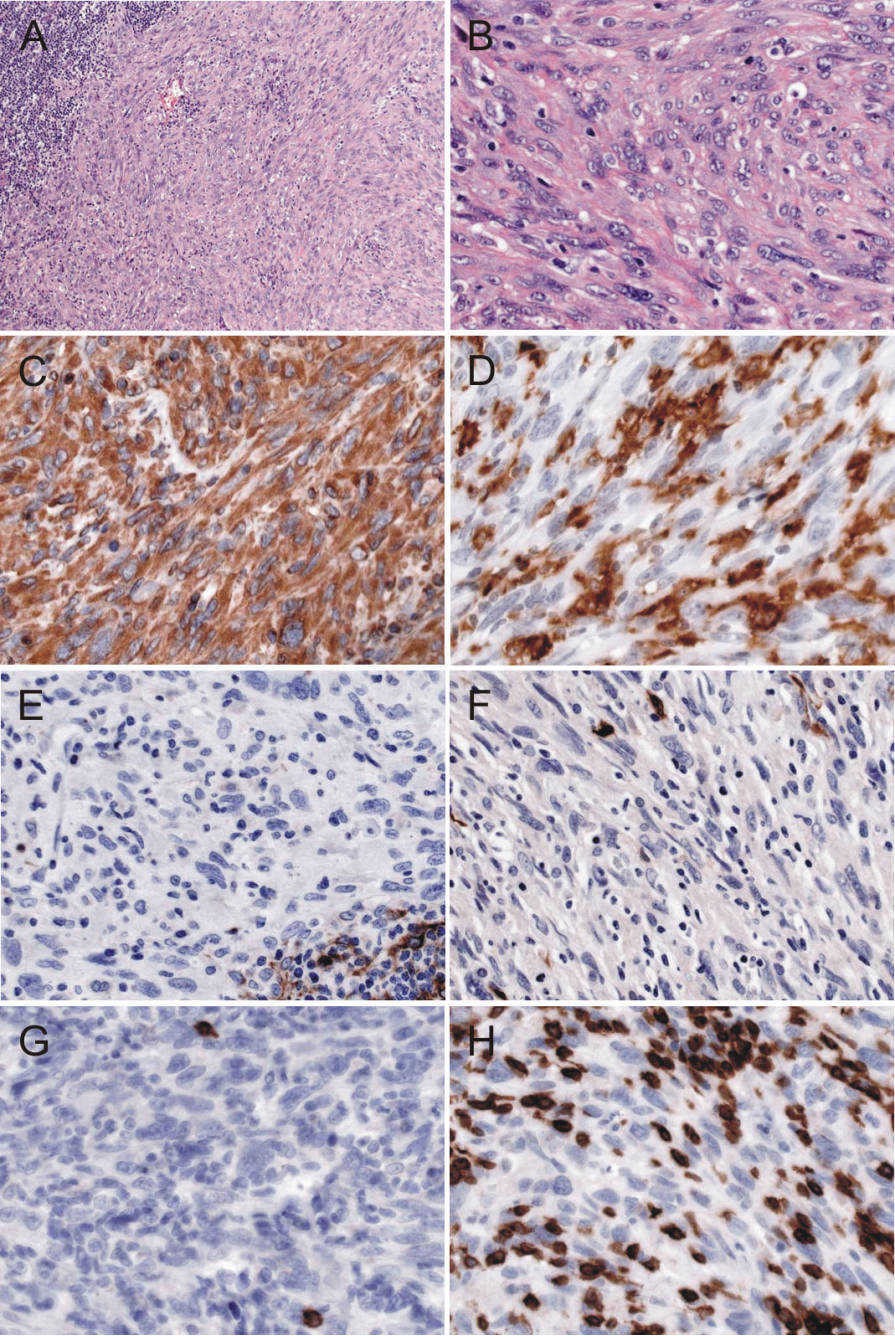
**Morphology and immunohistochemical stainings**: (**A**) H&E (original magnification 100×) and (**B**) H&E (original magnification 400×) show a spindle cell proliferation in an intraparotideal lymph node with moderate cellular pleomorphy (**C**) Vimentin (original magnification 400×) and (**D**)HLA-DR (original magnification 400×) were strongly expressed by the tumor cells. (**E**) Clusterin (original magnification 400×) and (**F**) S100 protein (original magnification 400×) were negative in the tumor cells. (**G**)CD20 showed only very few reactive small B-cells (original magnification 400×) and (**H**) CD3 some reactive T-lymphocytes (original magnification 400×). Virtual Slides: http://diagnosticpathology.slidepath.com/dih/webViewer.php?snapshotId=1316777507,  http://diagnosticpathology.slidepath.com/dih/webViewer.php?snapshotId=1316777681,  http://diagnosticpathology.slidepath.com/dih/webViewer.php?snapshotId=1316777716,  http://diagnosticpathology.slidepath.com/dih/webViewer.php?snapshotId=1316777754,  http://diagnosticpathology.slidepath.com/dih/webViewer.php?snapshotId=1316777781

The specimen consisted of parotid gland tissue containing a single firm, nodule 2.3 cm in diameter. The cut surface was soft and homogeneous light-grey. Microscopic examination revealed a spindle cell neoplasia in an intraparotideal lymph node. Tumor cells featured moderate nuclear pleomorphism, bulked chromatin and a clearly perceptible nucleolus (Figure 2A, B). Furthermore an elevated mitotic rate (up to 27 mitoses per 10 high power fields) was found.

### Immunohistochemical Findings

To further characterize the histogenesis of this tumor, immunohistochemical stainings were performed. The tumor cells were positive for vimentin, fascin and HLA-DR (Figure 2B, D). In contrast, the neoplastic cells were negative for all epithelial, melanocytic, lymphoid, histocytic and follicular dendritic cell markers used. The proliferation, as assessed by Mib1 index was 40-50%. The results of the immuohistochemical stainings are shown in table 1.

### Electron Microscopic Findings

**Figure 3:**
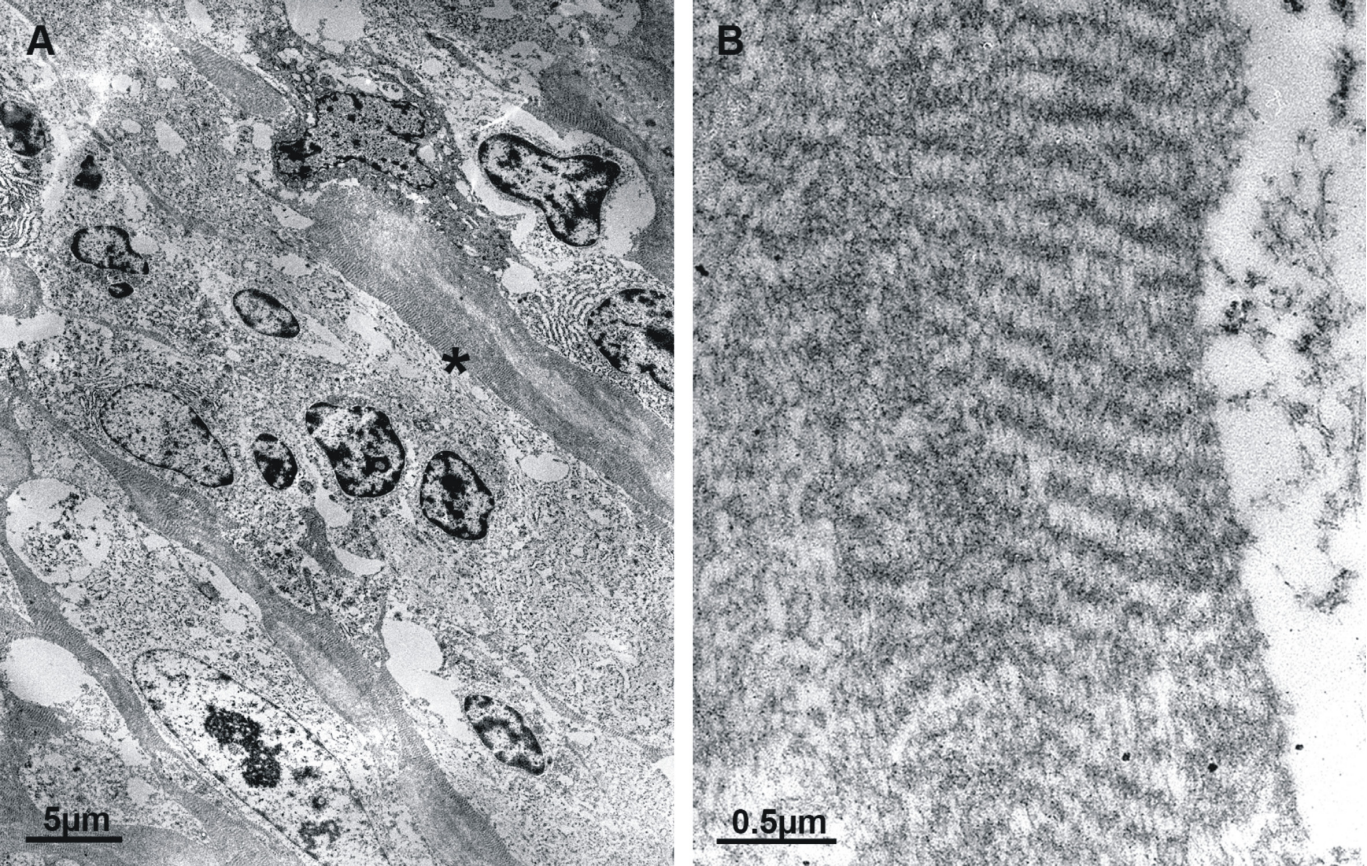
**Electron microscopy (EM)**: Most prominent feature was the large number of slender long-spacing collagen fibers dividing the tissue in many compartments (**A**). Fibrous long-spacing collagen the period length of which was in the order of 200 nm in this case (**B**), is not described in the literature to be specific for any disease, but has been found in a variety of normal and pathological tissues. Well-formed desmosomes or Birbeck granules were not found in this case.

The electron microscopical aspect of the tissue was characterized by cells which were devoid of characteristic structures as desmosomes or Birbeck granules. There was a certain heterogeneity of nuclei concerning their amount of heterochromatin. However, the cytoplasm of the cells (size, content of mitochondria or endoplasmatic reticulum) was less well differentiated (Figure 3A). The most prominent finding was the large number of slender long-spacing collagen fibers dividing the tissue in many compartments (Figure 3A). Fibrous long-spacing collagen the period length of which was in the order of 200 nm in this case (Figure 3B), is not described in the literature to be specific for any disease, but has been found in a variety of normal and pathological tissues. Therefore, it cannot be used as a pathognomonic marker here.

In summary, the morphological and immunophenotypic features were consistent with a dendritic cell tumor, not otherwise specified (NOS) originating in an intraparotideal lymph node. A differentiation towards a follicular or interdigitating dendritic cell sarcoma could not be demonstrated.

## Conclusions

Dendritic cell sarcomas (DCS) are rare neoplasms arising from antigen-presenting cells of the immune system [2]. DCS are being recognized with an increasing frequency. These tumors can occur, where dendritic cells are located. One of the most common manifestation sites are the head and neck region. Under-recognition of this entity may contribute to its rarity. The WHO subclassifies DCS into five groups [2]: Langerhans cell histiocytosis, Langerhans cell sarcoma, interdigitating dendritic cell sarcoma (IDCS), follicular dendritic cell sarcoma (FDCS) and other rare and less well classifiable dendritic cell tumors like fibroblastic reticular cell tumors, indeterminate dendritic cell tumors and dendritic cell tumors, not otherwise specified (DCT, NOS) [2]. FDCS is the most common subtype followed by IDCS.

The clinical course of this neoplasia is similar to that of soft tissue sarcomas [3,6]. Since only 306 cases of DCS have been reported worldwide (table 2), there is no general consensus on the treatment of this rare disease. Surgery has remained the gold standard in localized disease, but a recurrence rate up to 50% has been reported [5]. Alternatively some DCS have also been treated additionally by radiotherapy and or chemotherapy [7]. For our patient we chose the option of surgical therapy and he is well and free of disease for 2 years after surgery now.

**Table 2:** Overview of dendritic cell sarcoma case reports.

	FDCS	IDCS	
1986 - 4/2007 *			

Nodal	77	33	110
Extranodal	52	22	74

5/2007 - 08/2011 [5,7,10-86]			

Nodal	68	14	82
Extranodal	33	7	40

	230	76	306

FDCS, follicular dendritic cell sarcoma; IDCS, interdigitating cell sarcoma. * Modified from [5]. A total of 145 nodal FDCS cases, 85 extranodal FDCS cases, 47 nodal IDCS cases and 29 extranodal cases have been reported until 08/2011. 2 cases FDCS and 2 cases of IDCS occured in the parotid gland.

For establishing the diagnosis a large number of immunohistochemical stainings and ultrastructural analyses has been performed. Other rare differential diagnosis of salivary glands, like lipomatous pleomorphic adenoma [8] or sebaceous adenoma [9], were excluded. Despite this extensive work-up a definite assignation to an established entity of dendritic cell tumors could not be achieved. Therefore a diagnosis of dendritic cell tumor, NOS was made.

In summary, we report on the clinical, pathologic, and immunohistochemical features of a dendritic cell tumor, NOS in an intraparotideal lymph node. Metastatic disease was ruled out by PET- and CT imaging. Recognition of this extremely rare tumor, and its distinction from more common malignant tumors of lymph nodes and salivary glands largely depends on judicious application of immunohistochemical and ultrastructural techniques. The precise histogenesis of these extraordinarily rare tumors remains to be established.

## Consent

Written informed consent was obtained from the patient for publication of this Case Report and any accompanying images. A copy of the written consent is available for review by the Editor-in-Chief of this journal.

## Competing interests

The authors declare that they have no competing interests.

## Authors' contributions

SPS SKP and RS designed the study, collected the clinical data and drafted the manuscript. HW carried out the electron microscopic analyses. FF and PA performed the histopathologic and immunohistochemical analyses and helped to draft the manuscript. All authors read and approved the final manuscript.
